# Mitochondrial inhibitors reveal roles of specific respiratory chain complexes in CRY-dependent degradation of TIM

**DOI:** 10.1038/s41598-024-77692-0

**Published:** 2024-10-30

**Authors:** Xiangzhong Zheng, Dechun Chen, Brian Zoltowski, Amita Sehgal

**Affiliations:** 1 Department of Neuroscience, University of Pennsylvania Perelman School of Medicine, Philadelphia, PA 19104 USA; 2 Department of Biology, Indiana University, Bloomington, IN 47405 USA; 3 Department of Chemistry, Southern Methodist University, Dallas, TX 75275 USA; 4 Howard Hughes Medical Institute, Chevy Chase, MD 20815 USA

**Keywords:** Circadian clock, Cryptochrome, Timeless, Mitochondria, Respiratory chain, Biochemistry, Cell biology, Molecular biology, Neuroscience, Physiology

## Abstract

Drosophila Cryptochrome (CRY) is an essential photoreceptor that mediates the resetting of the circadian clock by light. in vitro studies demonstrated a critical role of redox cycling of the FAD cofactor for CRY activation by light. However, it is unknown if CRY responds to cellular redox environment to modulate the circadian clock. We report here that the mitochondrial respiratory chain impinges on CRY activity. Inhibition of complex III and V blocks CRY-mediated degradation of TIMELESS (TIM) in response to light, and also blocks light-induced CRY degradation. On the other hand, inhibition of complex I facilitates TIM degradation even in the dark. Mutations of critical residues of the CRY C-terminus promote TIM degradation in the dark, even in the presence of complex III and V inhibitors. We propose that complex III and V activities are important for activation of CRY in response to light. Interestingly, we found that transcriptional repressor functions of Drosophila and mammalian CRY proteins are not affected by mitochondrial inhibitors. Together these data suggest that the two functions of CRY have different sensitivity to disruptions of the mitochondrial respiratory chain: one is sensitive to mitochondrial activities that enable resetting, the other is insensitive so as to sustain the molecular oscillator.

**Supplementary Information:**

The online version contains supplementary material available at 10.1038/s41598-024-77692-0.

## Introduction

Circadian timing systems in most organisms have evolved to cope with the day:night cycle on earth. While specific components differ among species, the basic framework of molecular circadian clocks is well conserved among eukaryotes. In a nutshell, such molecular clocks consist of an activator that drives the expression of a repressor, which in turn inhibits the activator. In Drosophila, the heterodimeric complex of Clock (CLK) and Cycle (CYC) proteins activates the transcription of repressor genes *period* (*per*) and *timeless* (*tim*). Accumulation of PER and TIM proteins leads to a heterodimeric complex that represses transcription of these two genes. A series of post-transcriptional and post-translational steps are regulated to generate temporal delays, which are essential to sustain a cycle and presumably also a ~ 24 h period^1^. This negative feedback loop thus maintains rhythmic activity of CLK-CYC, which in turn drives rhythmic expression of other target genes to regulate cellular physiology and metabolism.

For optimal function, such a timing mechanism needs to be synchronized with cyclic environmental cues, among which the light–dark cycle is the most prominent factor. In flies, the FAD-binding Cryptochrome protein functions as a blue-light photoreceptor to transmit environmental light:dark signals to the molecular circadian clock^2,3^, mainly through TIM^4–7^. Light-activated CRY has a higher affinity for TIM and it promotes TIM degradation by the proteasomal machinery^4–6,8–11^, such that TIM levels remain low during the day. After lights-off, TIM accumulates and binds to PER to protect it from degradation, so PER levels peak in the late night, followed by nuclear localization of PER-TIM and subsequent inhibition of CLK-CYC activity. Light at dawn promotes degradation of TIM, which allows a new cycle to begin^1^.

Light activation of CRY is believed to involve a redox reaction of the FAD cofactor, and it is postulated that photo-activation of CRY results in a conformational change that disengages its auto-inhibitory C-terminus from its photolyase homology region (PHR)^12–14^. However, current understanding of CRY photo-activation is based mainly on studies of light-induced FAD redox cycles and CRY conformational change assayed by trypsin digestion in vitro and by structure modeling^12,13,15–21^. It has been demonstrated that the proximal light-signaling event involves light-induced electron transfer from a series of conserved tryptophan residues (Trp-Triad) to oxidized FAD to generate a flavin anionic semiquinone FAD^•-^. Generation of FAD^•-^ then induces protonation of His378, which lies between the flavin and an FFW motif (Phe534, Phe535, Trp536) in the CRY C-terminal tail (CTT)^22^, that leads to release of the CTT from the PHR. Release of the CTT then exposes a protein–protein interaction surface that is involved in CRY-TIM binding in Drosophila S2 cells^23,24^, and verified by recent CryoEM structures^14^. It is still largely unknown how CRY biochemical kinetics are linked to TIM degradation in cells. Intriguingly, CRY mutants lacking the CTT are still functional as circadian photoreceptors in flies, albeit with reduced sensitivity^11,25^, suggesting that the dynamic fold/release of the CTT is not essential for its circadian function. In contrast, a *cry*^b^ mutant that is defective in FAD binding displays severe defects in circadian photo-responses, indicating that FAD binding to CRY is necessary for circadian photoreception^3,11,25^.

In addition to light, other environmental cues are able to entrain the molecular clock. Among them, food is a potent factor that entrains the molecular clock in some tissues. The molecular basis of food entrainment is not understood, but one possible mechanism is through changes of the cellular metabolic environment. In mammals, transcriptional activity of CLOCK-BMAL1, orthologs of CLK-CYC, is regulated by NAD^+^ levels^26–31^ and mammalian CRY is also regulated by AMP kinase (AMPK), suggesting that energy metabolism impinges on the circadian clock. However, transcriptional activity of mammalian CRY, which is a transcriptional repressor rather than a photoreceptor, or Drosophila CRY, which can also repress transcription in some tissues, is not known to be sensitive to mitochondrial respiration, which is the hub of energy metabolism.

Here we report roles of specific respiratory chain complexes in regulating CRY-mediated degradation of TIM. We found that inhibition of complex I by rotenone promotes TIM degradation, whereas inhibition of complex III and complex V blocks TIM degradation. We further identified crucial residues in the CRY C-terminus that mediate TIM degradation. However, despite the importance of mitochondrial respiration for the photoresponse of CRY, we found that it is not required for the transcriptional repressor functions of Drosophila or mammalian CRY. Thus, the mitochondrial respiratory chain is probably not the source of changes in cellular metabolism that regulates circadian transcriptional activity.

## Results

### Complex III and complex V inhibitors block light-dependent TIM degradation

**Fig. 1:**
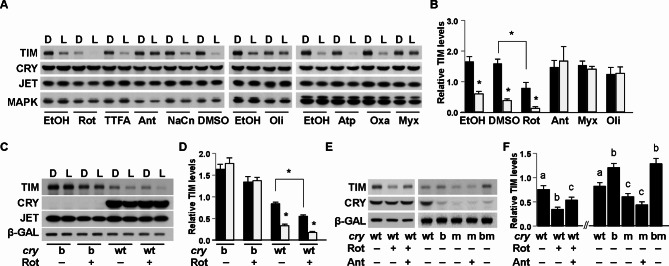
Mitochondrial inhibitors differentially affect stability of the TIM protein. Drosophila S2R + cells were acutely treated with vehicle or specific respiratory chain inhibitors in the dark, followed by continued darkness or light exposure. (**A**,**B**) Inhibitors of complex III (antimycin A, Ant; myxothyzol, Myx) and V (oligomycin, Oli) block light-dependent TIM degradation, whereas complex I inhibitor rotenone (Rot) promotes TIM degradation. No discernible effects were observed for complex II inhibitors TTFA, atpenin (Atp), oxaloacetate (Oxa), and complex IV inhibitor sodium cyanide (NaCn). Representative Western blots are shown in (**A**) and intensity of the TIM and MAPK loading control bands are quantified in (**B**). (**C**,**D**) Effect of rotenone on TIM stability requires CRY. In cells transfected with the mutant construct *cry*^b^, rotenone has minimal effect on TIM degradation. In contrast, rotenone promotes TIM degradation in the presence of CRY. Representative Western blots are shown in (**C**) and intensity of the TIM and b-GAL loading control bands are quantified in (**D**). (**E**,**F**) In cells expressing wildtype CRY, the effect of rotenone (2 μM) on TIM stability is partially rescued by antimycin A (2 μM); In contrast, TIM is destabilized in cells expressing a CRY that lacks its C-terminus (CRY^m^), and antimycin A does not block the effect of CRY^m^ on TIM. Black filled bars denote darkness, and open bars denote light conditions in B and D. Error bar denotes standard error of the mean, *n* > 3. Asterisk denotes significant difference (*p* < 0.05) by Student’s *t*-test. Different letters above the bars denote significant differences between bars on either the left or the right side of panel F (one-way ANOVA and post hoc Tukey test, *p* < 0.05). Full-length blots are presented in Supplementary Figures S2, S8, S9.

To determine the role of mitochondrial respiration in regulating CRY-dependent degradation of TIM, we used a well-established Drosophila S2 cell assay in which these cells are transfected with plasmids encoding CRY, TIM and JETLAG (JET), the E3 ligase that targets TIM for degradation in response to light^9^. We treated transfected cells with specific inhibitors of complex I, II, III, IV or V for 1 h in the dark and then exposed them to a light pulse (20 min). In this timeframe, TIM is degraded by light in a CRY and JET-dependent fashion, but CRY is minimally degraded. Multiple complex II inhibitors (2-thenoyltrifluoroacetone, TTFA; atpenin A5, Atp; oxaloacetate, Oxa) and a complex IV inhibitor (sodium cyanide, NaCN) did not have a discernable effect on light-dependent degradation of TIM at the concentration (10 μM) tested (so they were not included in later experiments). Complex III inhibitors, antimycin A (Ant) and myxothiazol (Myx), as well as a complex V inhibitor, oligomycin (Oli), blocked TIM degradation by light. In contrast, the baseline TIM level was dramatically reduced in cells treated with complex I inhibitor rotenone (Rot) both in darkness and light conditions (Fig. 1A,B). This effect was largely blocked in cells expressing a mutant CRY that is defective in FAD binding, CRY^b^ (Fig. 1C,D), suggesting that the effect of rotenone on TIM is at least partially CRY-dependent. We speculate that low-level endogenous expression of CRY in S2 cells^10,32^ contributes to the small (but not statistically significant) effect of rotenone on TIM in the dark in cells transfected with a *cry*^b^ construct.

## Inhibition of complex III does not block the effect of constitutively active CRY on TIM in the dark

Rotenone destabilizes TIM in darkness, a phenotype reminiscent of the effect of a constitutively active CRY mutant lacking the C-terminus (CRYΔ). When overexpressed in flies, CRYΔ mimics the effect of light to reduce TIM levels^25^. We found that in S2 cells also, TIM levels were reduced in the presence of a mutant CRY^m^^11^ that lacks the C-terminus (Fig. 1E,F). Interestingly, the complex III inhibitor, antimycin A, partially rescued rotenone-mediated reduction of TIM in darkness (Fig. 1E,F, Fig S1), but it had no effect on CRY^m^-mediated TIM degradation. Similar effects were observed for the complex V inhibitor oligomycin (Fig S2). In contrast, TIM levels were restored when deletion of the C-terminus was coupled with *cry*^b^ mutation of the flavin binding residue^2,3^ (see *cry*^bm^ data in Fig. 1E,F), indicating that FAD binding is required for the activity of CRY^m^. Additionally, these data demonstrate that removing the C-terminus of wildtype CRY is sufficient to override the effect of complex III inhibitor on TIM.

### Specific mutations of CRY in the C-terminus promote TIM degradation

**Fig. 2:**
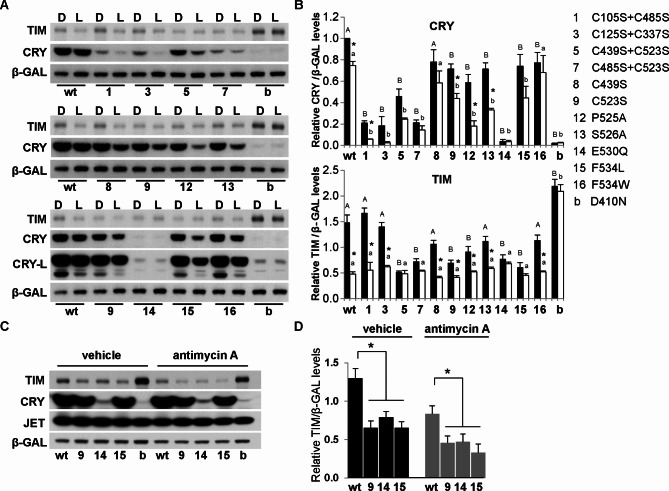
CRY constructs carrying specific mutations of cysteine and the C-terminus tail differentially affect TIM stability. (**A**) Mutating a series of cysteine residues reveals that the C523S mutation in CRY destabilizes TIM in the dark (top and middle panels). While most constructs carrying mutated residues in the CRY C-terminus have partial effects on TIM stability in the dark, E530Q and F534L have dramatic effects on light-dependent degradation of TIM (middle and bottom panel). Quantification of TIM and loading control band b-GAL is shown in (**B**). Black filled bars denote darkness, and open bars denote light conditions. Error bars denote standard error of mean, n = 3. Asterisk denotes significant difference between dark and light (*p* < 0.05 by Student’s *t*-test); different upper-case letters denote significant difference between wildtype and mutant constructs in dark, whereas different lower-case letters denote significant difference between wildtype and mutant constructs in light (one-way ANOVA, post hoc Tukey test, *p* < 0.05);. (**C**,**D**) Effect of C523S, E530Q and F534L on TIM levels in dark is not blocked by a mitochondrial complex III inhibitor antimycin A. A representative Western blot is shown in (**C**) and quantification of TIM and loading control band b-GAL is shown in (**D**). Error bar denotes standard error of mean, *n* = 3. Asterisk denotes significant difference by Student’s *t*-test, *p* < 0.05. Full-length blots are presented in Supplemental Figure S4 and S10.

One current model posits that light triggers conformational changes of CRY within a linker region connecting the N-terminal PHR to the species-specific CRY CTT (Fig S3). These conformational changes are believed to be coupled to the reduction of an FAD cofactor resulting in release of the CTT^12^. It is possible that other mechanisms mediate CRY CTT release, including other redox regulated pathways. Because cysteine residues are known to function as redox sensors to regulate protein functions^33,34^, we mutated a series of cysteine residues to determine their potential roles in regulating CRY activity. Interestingly, C105S-C485S and C125S-C337S double mutations had a dramatic effect on CRY stability in the dark and even more so in the presence of light. However, they had no significant effect on TIM stability or light-induced TIM degradation. On the other hand, C439S-C523S and C485S-C523S mutants destabilized both CRY and TIM and prevented any further reduction of TIM by light (Fig. 2A,B). We further tested C439S and C523S single mutants and found that C439S had minimal effect on CRY and TIM, but C523S reduced CRY and TIM stability, while still promoting their light-induced degradation. TIM levels under light conditions were similar between cells transfected with C523S mutants and wildtype CRY, perhaps indicating “floor” effects in this assay.

Although mammalian CRY1 has an inter-molecular disulfide bridge^35^, cysteine residues in Drosophila CRY are not involved in disulfide bonding^16,22,36^. Rather, the crystal structure of Drosophila CRY indicates a crucial positioning of the C523 residue within the turn motif linking the PHR and CTT^16,36^ (Fig S3). In this manner, C523S substitution may facilitate the release of the CTT, thus rendering CRY constitutively active.

We further constructed mutants to identify critical residues in the C-terminus that regulate CRY activity. The interaction between the FAD binding pocket and the turn motif involves a stacking interaction between Trp422-Pro525 (Fig S3). Interestingly, although P525A had only a small effect on CRY stability in the dark, it greatly promoted light-dependent degradation of CRY (Fig. 2A,B). On the other hand, the CRY^P525A^ variant destabilized TIM in the dark, and light further degraded TIM to a level similar to that in cells transfected with wildtype CRY (Fig. 2A,B).

The FAD binding pocket of CRY also interacts with the turn motif through H-bonding interaction between Ser526-Glu530 (Fig S3). Previous studies indicated that an S526A variant significantly reduces the light-induced trypsin-sensitivity of CRY in vitro^16^ and CRY-TIM interactions in a yeast-2-hybrid assay^37^. In contrast, we found that the CRY^S526A^ mutant was slightly less stable in the dark, and more sensitive to light-induced degradation in S2 cells (Fig. 2A,B). However, the CRY^S526A^ variant did not significantly affect TIM stability.

To further evaluate the role of the turn motif we mutated a conserved site (E530Q) that would retain H-bonding capabilities, but specifically alter local charge. Introduction of a Gln residue at position 530 destabilized CRY to a level similar to CRY^b^ (Fig. 2A,B), indicating that a negatively charged residue at this position is required to stabilize CRY. Even at these low levels, CRY^E530Q^ caused TIM degradation in the dark and no further reduction occurred upon light treatment (Fig. 2A,B). Such effects on TIM are reminiscent of the CRY mutants lacking the C-terminus^11,25^. Combined, these CRY variants indicate that the turn motif plays a critical role in stabilizing PHR-CTT interactions to enable CRY stability and regulate CRY’s activity towards TIM.

**Fig. 3:**
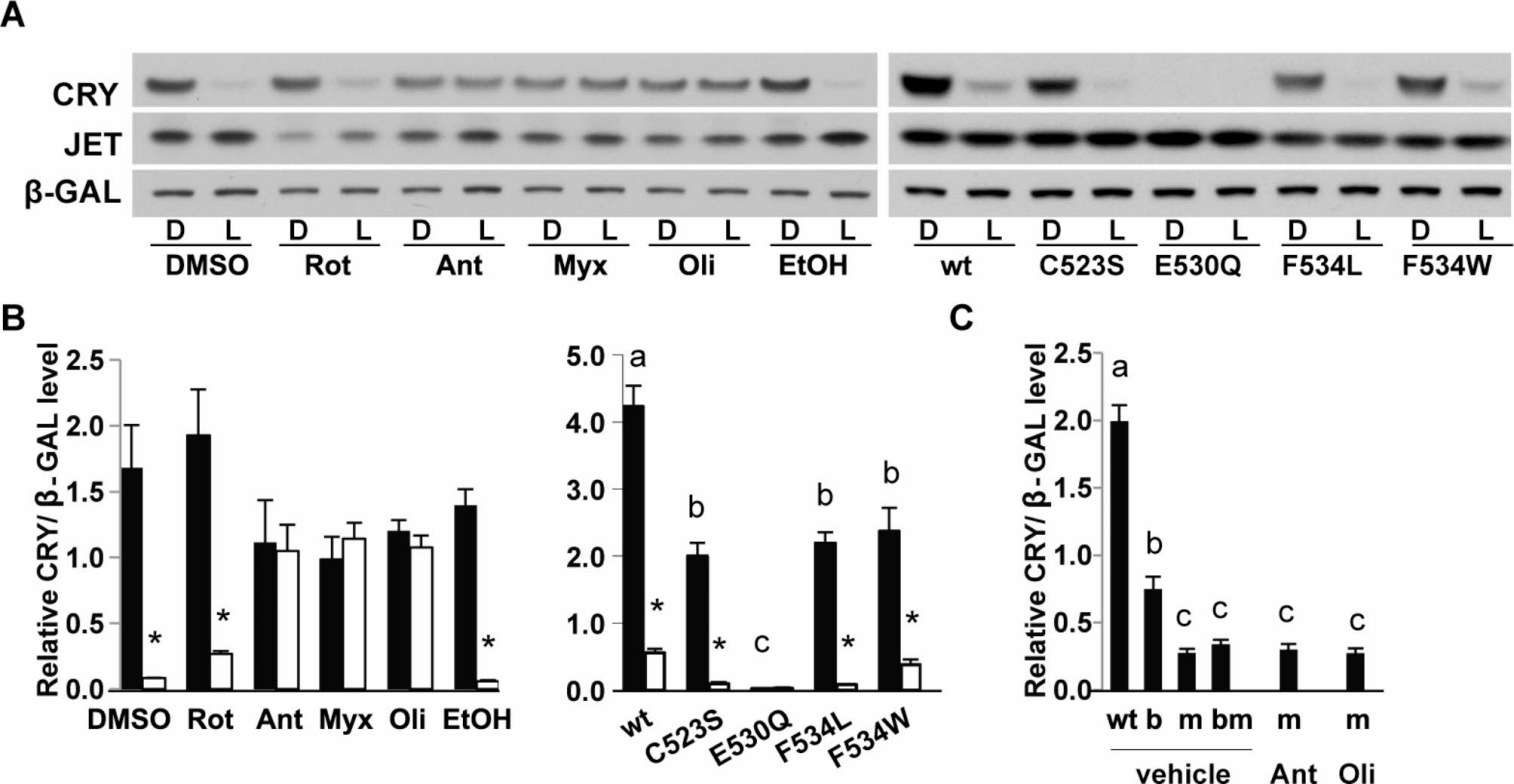
Effect of mitochondrial inhibitors and C-terminus mutations on CRY stability. (**A**) Left Panel: prolonged (2 h) light exposure effectively degrades CRY proteins in S2 cells treated with vehicle (DMSO, EtOH) or rotenone (Rot), whereas complex III (Ant, Myx) and V (Oli) inhibitors block light-dependent CRY degradation. Right panel: compared to wildtype (wt) CRY, levels of C523S, E530Q, F534L and F534W mutant proteins are reduced, and light exposure further destabilizes C523S, F534L and F534W proteins. (**B**) Relative protein levels were quantified from more than 3 Western blots. Asterisk denotes significant difference between dark and light, by Student’s *t*-test, *p* < 0.05. (**C**) Complex III and V inhibitors do not block degradation of CRY^m^ (m) proteins. Error bar denotes standard error of the mean, *n* > 3. Different letters above the bar denotes significant difference (one-way ANOVA, post hoc Tukey test, *p* < 0.05). Full-length blots are presented in Supplemental Figure S11.

To determine how FAD chemistry is communicated to the CTT, we further examined residues near the FAD binding pocket for their effect on CRY and TIM stability. The proximal residue that may relay a signal for conformational change from FAD to the CTT is F534^16,36^ (Fig S3). Whereas a F534A variant was poorly expressed^12^, we found that expression levels of F534L and F534W were similar to each other, although lower than those of wildtype CRY (Figs. 2A,B, 3A,B). Interestingly, these two mutant proteins demonstrated substantial differences in their ability to influence TIM stability. F534L destabilized TIM in the dark to a level normally seen in light (Fig. 2A,B). On the other hand, the F534W mutant had a minimal effect on TIM levels in the dark, light-dependent degradation of TIM was also largely unaffected.

These data suggest that C523S, E530Q and F534L mutants are constitutively active, and thereby similar to CRY^m^ in promoting TIM degradation in the dark. Consistent with the idea that the CTT regulates CRY stability, we found that the C523S, E530Q, F534L variants of CRY are less stable (Figs. 2, 3). Furthermore, antimycin and myxothiazol did not block their effects on TIM in the darkness (Fig. 2C,D, Fig S4 ).

### Complex III and V inhibitors block CRY degradation

As noted above, the assay conditions for TIM degradation experiments are not ideal to evaluate degradation of wildtype CRY, but prolonged illumination will also degrade CRY^2,10,11^. To test if inhibition of the mitochondrial respiration chain affects light-induced CRY activation and subsequent degradation, we treated S2 cells with mitochondrial inhibitors for 1 h in dark, followed by light-exposure for 2 h to maximize CRY degradation. Interestingly, CRY degradation was not significantly affected by rotenone treatment (Fig. 3), which promoted TIM degradation (Fig. 1), suggesting that complex I is relevant for CRY signaling to TIM, but not for CRY degradation. On the other hand, complex III and V inhibitors blocked light-induced CRY degradation (Fig. 3), similar to their effects on TIM (Fig. 1). These results suggest that light-induced CRY activation is impaired by these inhibitors (Fig. 3). Importantly, complex III and V inhibitors did not increase the stability of active CRY proteins lacking the C terminus, CRY^m^^2,3,11^ (Fig. 1E, Fig S2, Fig. 3C). These data are consistent with previous findings that CRY activation due to conformational changes involving the C-terminus is irreversible^38^.

Because C-terminus mutants C523S, E530Q and F534L were less stable than wildtype CRY under conditions we used to assay TIM stability and they were constitutively active toward TIM (Fig. 2), we also examined the effects of prolonged light on their degradation. While E530Q was equally unstable in darkness and light conditions, C523S and F534L were dramatically degraded by light, more so than a shorter light exposure (20 min) (Figs. 2, 3).

### The transcriptional repressor function of CRY is not sensitive to mitochondrial inhibition

**Fig. 4:**
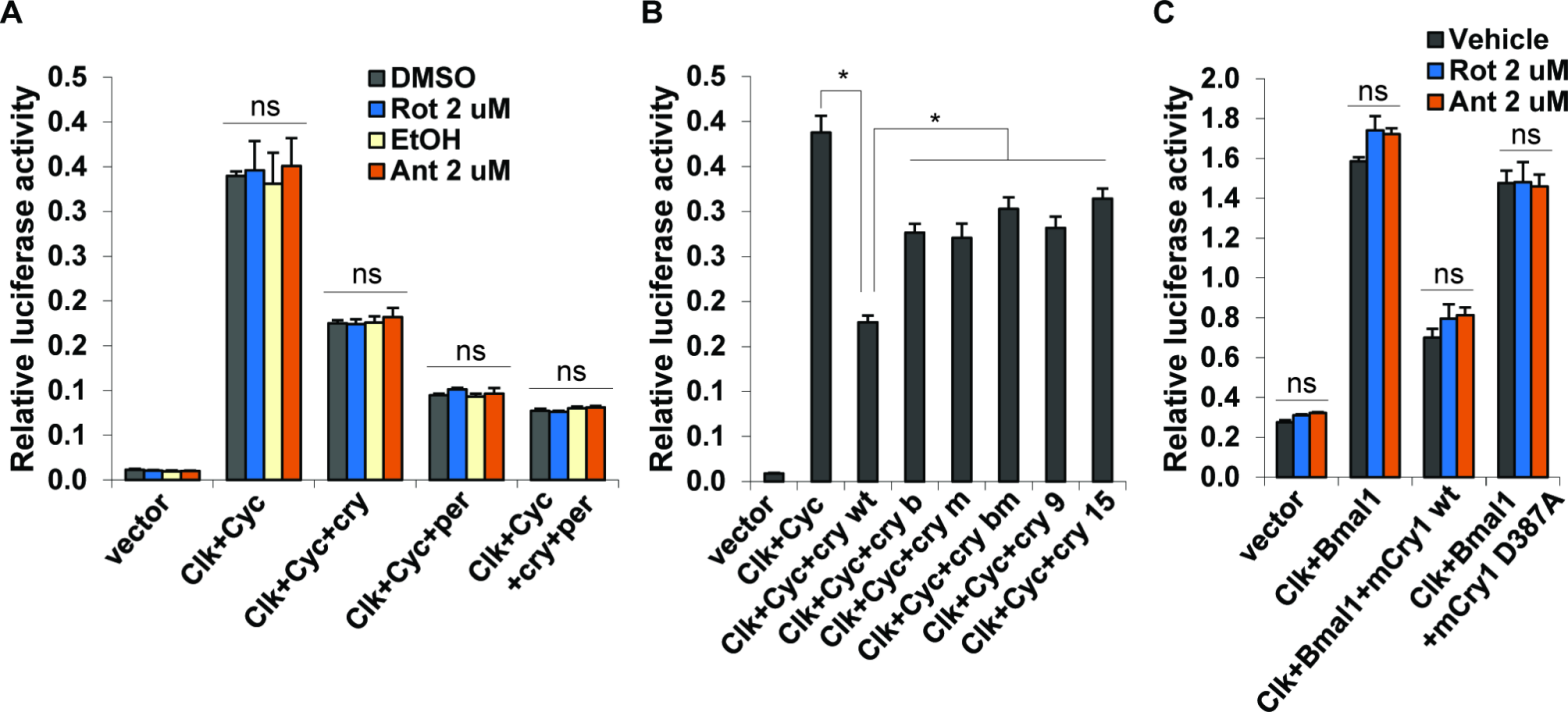
Effect of mitochondrial inhibitors on the transcriptional repressor activity of CRY. (**A**) In HEK 293 T cells, CLK-CYC activates *per*-*luc2p* transcription, which is partially repressed by Drosophila CRY or PER. Rotenone (Rot) and antimycin (Ant) have no effect on *per*-luciferase activity. ns denotes no significant difference by one-way ANOVA. (**B**) In HEK 293 T cells, CRY mutants are less effective than wildtype CRY in repressing *per-luc2p* reporter activity, most likely due to their lower expression levels. Error bar denotes standard error of mean, *n* = 3. Asterisk denotes significant difference (between wt and each mutant group) by Student’s *t*-test, *p* < 0.05. (**C**) In S2 cells, CLOCK-BMAL1 activates *per1*-d*luc* transcription, which is repressed by wildtype mCRY1 (wt). The mCRY1 D387A mutation (equivalent of Drosophila CRY^b^) abrogates such repression. Mitochondrial inhibitors rotenone (Rot) and antimycin (Ant) have no effect. ns denotes no significant difference by one-way ANOVA.

Drosophila CRY is well-characterized as a circadian photoreceptor, but it also functions as a transcriptional repressor in some cellular environments^39,40^. To test if the repressor function of Drosophila CRY (dCRY) is responsive to mitochondrial inhibition, we transfected d*cry* into HEK 293 T cells as previously reported^40^. Surprisingly, we found that complex I and complex III inhibitors had no effect on CRY mediated repression of CLK-CYC transcriptional activity (Fig. 4A). In addition, mutations that have opposing effects on CRY’s modulation of TIM (Fig. 2) – namely those that render CRY constitutively active (CRY^m^, CRY^C523S^, CRY^F534L^) and those that inactivate it (CRY^b^, CRY^bm^) – had similar effects on CLK-CYC activity (Fig. 4B). The difference between wildtype CRY and mutant CRYs is probably due to their different expression levels (Fig S5). These data suggest that transcriptional repressor activity of Drosophila CRY is not sensitive to changes in mitochondrial respiration.

Recent studies showed that mammalian CRY1 can be redox sensitive in certain cellular environments^19,41,42^. To test if mitochondrial inhibitors affect the repressor function of mCRY1, we assayed mCRY1 repression of *per*-luciferase reporter activity as reported^41^. While a D387A mutation (an equivalent of the Drosophila *cry*^b^ mutation) relieved mCRY1 repression of CLOCK-BMAL1 activity as reported^41^, complex I and complex III inhibitors rotenone and antimycin had no effect on mCRY1 mediated repression of CLOCK-BMAL1 activity in S2 cells (Fig. 4C).

## Discussion

Circadian clocks are thought to have evolved to maximize organism fitness in cyclic environments, in particular the light:dark cycle generated by the rotation of the earth. Drosophila CRY is well established as a flavin-based circadian photoreceptor^2,10,11,43^, such that a mutation in the FAD binding domain of CRY renders it blind to light^2,3^. When purified from insect cells, dCRY contains oxidized FAD, which can be reduced by light^19^. One model posits that FAD reduction by either light or a reducing agent induces a release of the self-inhibitory CTT to promote TIM degradation^12,36,44,45^. However, how FAD chemistry induces conformational changes remote from the FAD active site is still debated. Furthermore, mutations of the Trp triad that block all known or presumed intra-protein electron transfers do not affect dCRY phototransduction as measured by light-induced proteolysis of dCRY and dTIM in Drosophila S2 cells^15^. Thus, it is still largely unknown if and how cellular environment regulates CRY chemistry, or how it impacts the clock.

In this report we show that inhibition of complex III and V blocks light-dependent TIM degradation, whereas inhibition of complex I by rotenone promotes TIM degradation. We further demonstrate that complex III and V inhibitors block wildtype CRY activation, but have no effect on constitutively active CRYs that harbor C-terminal mutations, or lack the C-terminus. These findings suggest that CRY activation before irreversible conformational change^38^ is modulated by the mitochondrial respiratory chain. However, these effects of the mitochondrial respiratory chain are restricted to the function of CRY as a photoreceptor, with no impact on the transcriptional repressor activity of CRY.

### Role of the mitochondrial respiration chain in CRY-mediated TIM degradation

The mitochondrial inhibitors used in this study either interfere with electron transport, or disrupt the ATP synthase (complex V). Because of the predominant role of mitochondria in providing ATP for cellular processes, it is possible that impairment of ATP production underlies the effect of mitochondrial inhibitors. However, previous studies in *Neurospora* have demonstrated that reduced ATP level is not directly correlated with effects on the circadian clock, as complete depletion of ATP by cyanide and antimycin A did not cause a significant phase shift^46^. We found that in Drosophila S2 cells, complex I and complex III/V inhibitors, all of which are known to reduce intracellular ATP levels, had opposite effects on TIM stability. For instance, combined treatment with complex I inhibitor (rotenone) and complex II inhibitor (TTFA), which are predicted to deplete ATP, actually destabilized TIM in a dosage-dependent manner (Fig S6A). Furthermore, combined treatment with rotenone and a glycolytic inhibitor 2-deoxyglucose, which together also effectively deplete intracellular ATP^47^, did not block light-dependent TIM degradation. Thus, loss of ATP did not block the TIM light response in the above cases although a low dose (0.1 uM) of complex III inhibitor (antimycin) was sufficient to block light-dependent TIM degradation (Fig S6B). The differential effects of mitochondrial inhibitors on CRY-mediated TIM degradation in S2 cells thus imply that factors other than ATP are involved in this process.

Complex I and complex III are the major sites of redox reactions in living cells. All inhibitors used in this study are known to increased ROS production^48^ and Ca[2 +] influx^49^, it is unknown if there are distinct reactive oxygen species and mitochondrial metabolites or differences in Ca[2 +] dynamics that affect CRY activity via similar or distinct mechanisms. It is possible that mitochondrial flavin is directly involved in signaling. FAD traffic across the mitochondrial membranes has been reported in yeast and mammals^50,51^ and some CRY species are expressed in cytosol and mitochondria^52,53^. We note that CRY is ubiquitously expressed in the cytoplasm, but it also appears to localize to mitochondria in S2 cells (data not shown), suggesting that CRY may shuttle between mitochondria and cytoplasm. However, the communication between the mitochondrial respiratory chain and CRY likely involves other unknown intermediates.

### C-terminal residues regulate CRY activity

Cysteine residues are known to sense cellular environment redox status. Alternatively, cysteine residues may be involved in relaying FAD chemistry to the TIM binding surface. Given the link between mitochondrial signaling and CRY function we examined the role of sulfur containing residues in mediating effects of cellular redox states on CRY. We identified several potential disulfide bond-forming cysteine pairs and mutated them to serine. Interestingly, we found that while C105S-C485S and C125S-C337S mutants are less stable, they are fully functional in promoting light-dependent degradation of TIM. On the other hand, C439S-C523S and C485S-C523S mutations destabilize CRYs, and also promote TIM degradation in darkness (Fig. 2A,B). Although purified Drosophila CRY proteins do not seem to form intra-CRY disulfide bonds^16,22,36^ like those in mammalian CRY1 (Cys363 and Cys412)^35^, it is unknown if such bonding exists in vivo, or if these cysteine residues form disulfide bond with other proteins, such as the inter-molecular disulfide bonding between mCRY1 and mPER2^35^.

Analysis of CRY structures and in vitro trypsinolysis experiments identified several surfaces that could mediate allosteric changes in CRY structure. Cys337 and Cys523 are in close proximity to the CTT and FAD binding pocket. The turn motif is further stabilized by insertion of C523 into a hydrophobic (sulfur-rich) cleft (Fig S3 C, D). In this regard C523 is ideally positioned to mediate signal communication between the FAD active site and the CTT. Mutation of these residues (C337A and C523A) differentially affects FAD photochemical kinetics and CRY proteolysis (assayed by trypsinolysis) in vitro: whereas a C337A variant increased proteolysis after 30–60 min of light exposure, a C523A variant had only a small effect on CRY proteolysis^16^. In contrast, we found that a C523S mutation destabilizes CRY in darkness, and it also promotes light-induced CRY degradation. Furthermore, a C337S mutation (together with a C125S mutation) destabilizes CRY in S2 cells (Figs. 2, 3). The C523S mutant promotes TIM degradation in the dark, while the C125S-C337S mutant has no effect on TIM degradation both in dark and in light (Fig. 2A,B). These data suggest that CRY proteolysis can be decoupled from its photo-signaling to TIM.

Examination of possible allosteric pathways linking CRY chemistry to the CTT identified a short turn motif that couples C-terminal elements and the FAD binding pocket. Here, a strong H-bonding interaction between E530 and S526 is presumed to be important for CRY stability and signaling. Structural analysis suggests that S526 constitutes an essential hinge residue gating tail release (Fig S3). Thus a S526A mutation presumably affects tail opening. It was previously reported that the S526A mutation significantly reduced light-induced trypsinolysis of CRY in vitro^16^. In addition, E530P and S526A variants also reduced CRY and TIM interactions in yeast-2-hybrid experiments^54^. In contrast, we found that the S526A mutant increases light-induced degradation of CRY in S2 cells, but it has only a small (but not statistically significant) effect on TIM in the dark (Fig. 2 A-B). The discrepancy in these observations is likely due to different assay conditions.

To better understand conflicting data regarding the CRY turn motif, we further examined E530 in a cellular context and focused on a subtle mutation (E530Q) to only affect charge, but not abolish H-bonding interactions. CRY E530Q is extremely unstable (Fig. 2A,B), indicating that a negatively charged residue is required at this position for CRY stability. This mutant behaves like a constitutive CRY that lacks the C-terminus to promote TIM degradation in the dark. Thus, residues in the turn motif gate CRY function and dictate relative stability of both CRY and TIM.

Whereas our data suggest that the turn motif is directly involved in CRY regulation, the proximal residues capable of sensing chemical changes within the FAD-binding domain are F534 and W422, the latter of which anchors the turn motif, the CTT and a coiled-coil helix through possible H-bonding between E530 and S526 (Fig S7). Introduction of a Trp residue at position 534 places an electron-donor within 4.4 Å of FAD that π-stacks with W422 and renders CRY slightly more active in the dark, but still promotes light-stimulated signaling (Figs. 2,3). Interestingly, introduction of a Leu residue at 534 maintains a hydrophobic pocket that is not capable of direct electron-transfer, but light-dependent degradation of CRY is largely unaffected. Also, CRY-mediated TIM degradation is increased in the dark (Fig. 2A,B). It is possible that W422 functions as an electron donor in CRY in vivo. In this regard, F534L substitution may promote electron transfer from W422 to FAD to generate the FAD semiquinone and a W422 cation radical (W422 +). Deprotonation of W422 + by E530 could alter interactions within the CTT so as to maintain CRY stability. In this scenario, an E530Q variant that retains H-bonding functionality, but lacks the ability to deprotonate W422 + may lock CRY in an active but unstable state. Such a mechanism would implicate E530 in direct signal propagation following photoreduction of FAD.

Finally, it should be noted that while C-terminal mutations C523S and F534L reduce levels of TIM and CRY, they do not eliminate photosensitivity as these mutant CRY molecules are still degraded in response to light. Light-induced degradation of TIM is not as evident, perhaps due to a floor effect given that dark levels are also low. Light responsiveness of C terminal mutants is consistent with previous findings^11^ that CRY^m^ mutants, which lack the C terminus, are able to entrain the Drosophila clock, albeit weakly.

### Metabolic signaling to the circadian clock

It was reported that human CRY1 can be reduced by light when expressed in insect cells^19,42^, suggesting that mammalian CRY1 is redox sensitive. Interestingly, a D387A mutation in the FAD binding motif relieves the repression of CLOCK-BMAL1 by CRY (Fig. 3 and^41^). However, studies using other mutations of the putative FAD binding motif suggest that D387A affects transcriptional repression by disrupting the acidic surface region rather than FAD binding^16,41^. This would suggest that unlike photoreception by Drosophila CRY, repression of CLOCK-BMAL1 mediated transcription by mammalian CRY does not require an FAD redox reaction. Consistent with this finding, we found that mitochondrial inhibitors do not affect repressor functions of dCRY or mCRY1. These data suggest that CRY evolved two distinct mechanisms to regulate the circadian clock: one is sensitive to mitochondrial activities that enable resetting, the other is insensitive so as to sustain the molecular oscillator.

Cellular redox regulation of the circadian clock has been reported in other organisms. In cyanobacteria^29^ the light-sensitive protein LdpA acts as a redox sensor of cellular metabolic status to interact with the core clock machinery^29^. In mammals, the redox state of NAD regulates the circadian clock^30,31^. The *Neurospora oli*^r^ mutant and other strains with mutations in mitochondrial proteins, display a shorter circadian period that is correlated with elevated levels of total mitochondrial protein mass^55,56^. Our Drosophila S2 cells data suggest a common role of mitochondria in regulating the circadian clock, but it is likely that specific mechanisms differ between species and cell types.

## Materials and Methods

### Molecular cloning

To generate mutant *cry* constructs, pIZ-*myc-cry* or pIZ-*cry-V5* was used as template for site-directed mutagenesis by using the QuikChange Lightning Multi kit (Agilent technologies, Santa Clara, California). All constructs were verified by sequencing.

### Cell culture and treatments

Drosophila S2R^+^ cells (obtained from the Drosophila RANi Screening Center at Harvard University) were transfected with the following plasmids: pIZ-*tim*, pIZ-*myc-cry* or pIZ-*cry*-V5^57^, pIZ-Flag-*jet*^9^, and pAc-β*Gal*-V5. Cell culture plates were wrapped with double-layer aluminum foil to prevent light exposure. After 48 h, cells were treated with vehicle or specific mitochondrial inhibitors (10 uM except indicated in figures) in the darkroom for 1 h before light exposure of 20 min at 2000 lx light intensity, followed by 40 min incubation in darkness, then processed for cell lysis and Western blotting. For the CRY degradation assays, cells were treated with vehicle or inhibitors for 1 h in the darkroom before light exposure of 2 h at 2000 lx intensity. Dark control samples were wrapped in double layer foil.

To prepare stock solutions, ethanol was used for these inhibitors: Antimycin A, Oligomycin; DMSO was used for: Rotenone, Myxothiazol, TTFA, Atpenin A5, Apocyin; water was used for: Oxaloacetic acid, Sodium cyanide.

### Western blotting and image analysis

Cell culture medium was removed and cells were harvested into Eppendorf tubes with PBS buffer. After a brief spin, cell pellets were re-suspended with passive lysis buffer (Promega, Madison, WI, USA). SDS loading buffer was added into the whole cell extracts and the samples were boiled for 5 min then centrifuged for 2 min. Supernatants were loaded into a Bis–Tris gel, followed by SDS PAGE separation. Proteins were blotted onto a nitrocellulose membrane followed by primary antibody incubation overnight at 4 °C. Rat anti-TIM (UPR41, 1:1000), mouse anti-MYC (clone 9E10, Roche Diagnostics GmbH, Mannheim, Germany), rabbit anti-Flag (1:1000), rabbit anti-V5 (1:1000), mouse anti-HSP70 (1:20,000), rabbit anti-MAPK (1:1000) (Sigma, St. Louis, MO, USA) were used. HRP-conjugated secondary antibodies were used at 1:5000 dilution. Following ECL development (Pierce Biotechnology, Rockford, IL, USA), blots were exposed to X-ray film and developed to visualize protein bands. Films were scanned in UMAX scanner and bands were quantified by using Kodak Image Station. Polyclonal TIM antibodies (UPR41, UPR42) were produced in rats by immunization with His-tagged full length TIM protein.

### Transcriptional assay

Plasmids were transfected into Drosophila S2 cells (Gibco, ThermoFisher, Waltham, MA, USA) or HEK 293 T cells (ATCC, Manassas, VA, USA) in 96-well plates using Effectene (Qiagen, Valencia, CA, USA), according to the manufacturer’s protocol and cultured in darkness. For S2 cells, we subcloned h*Clock*, h*Bmal1* and m*Cry1* (a gift from John Hogenesch, University of Pennsylvania) into a pIZ vector. For HEK293T cells, *Clk*, *Cyc*, *cry*, and *per* in pCDNA3.1 vectors^40^ were used. 48 h after transfection, cells were treated with mitochondrial inhibitors (2 uM) or vehicles in a darkroom for 6 h. Cells were then harvested and luciferase activity was measured in a Victor 3 plate reader (PerkinElmer, Waltham, MA) by using Stop & Glow reagents (Promega, Madison, WI, USA). Firefly luciferase activity (pGL4.1-*Per1*-d*luc*, or pGL4-*per*-Ebox-*luc2p*) readings were normalized over pRL-CMV-Renilla-*luc* or p*Act*-Renilla-*luc* readings.

### Statistical analysis

Student’s *t*-test was used for comparisons between two groups. One-way ANOVA and post hoc Tukey test were used for comparisons among three or more groups.

## Electronic supplementary material

Below is the link to the electronic supplementary material.

Supplementary Material 1

## Data Availability

No datasets were generated or analysed during the current study.

## References

[1] Zheng, X. & Sehgal, A. Speed control: Cogs and gears that drive the circadian clock. *Trends Neurosci.* **35**(9), 574–585 (2012). 22748426 10.1016/j.tins.2012.05.007 PMC3434952

[2] Emery, P. et al. CRY, a drosophila clock and light-regulated cryptochrome, is a major contributor to circadian rhythm resetting and photosensitivity. *Cell* **95**(5), 669–679 (1998). 9845369 10.1016/s0092-8674(00)81637-2

[3] Stanewsky, R. et al. The cryb Mutation Identifies Cryptochrome as a Circadian Photoreceptor in Drosophila. *Cell* **95**(5), 681–692 (1998). 9845370 10.1016/s0092-8674(00)81638-4

[4] Hunter-Ensor, M., Ousley, A. & Sehgal, A. Regulation of the drosophila protein timeless suggests a mechanism for resetting the circadian clock by light. *Cell* **84**(5), 677–685 (1996). 8625406 10.1016/s0092-8674(00)81046-6

[5] Zeng, H. et al. A light-entrainment mechanism for the Drosophila circadian clock. *Nature* **380**(6570), 129–135 (1996). 8600384 10.1038/380129a0

[6] Lee, C. et al. Resetting the Drosophila clock by photic regulation of PER and a PER-TIM complex. *Science* **271**(5256), 1740–1744 (1996). 8596938 10.1126/science.271.5256.1740

[7] Ceriani, M. F. et al. Light-dependent sequestration of TIMELESS by CRYPTOCHROME. *Science* **285**(5427), 553–556 (1999). 10417378 10.1126/science.285.5427.553

[8] Naidoo, N. et al. A role for the proteasome in the light response of the timeless clock protein. *Science* **285**(5434), 1737–1741 (1999). 10481010 10.1126/science.285.5434.1737

[9] Koh, K., Zheng, X. & Sehgal, A. JETLAG resets the drosophila circadian clock by promoting light-induced degradation of TIMELESS. *Science* **312**(5781), 1809–1812 (2006). 16794082 10.1126/science.1124951 PMC2767177

[10] Lin, F.-J. et al. Photic signaling by cryptochrome in the drosophila circadian system. *Mol. Cell. Biol.* **21**(21), 7287–7294 (2001). 11585911 10.1128/MCB.21.21.7287-7294.2001 PMC99903

[11] Busza, A. et al. Roles of the two drosophila CRYPTOCHROME structural domains in circadian photoreception. *Science* **304**(5676), 1503–1506 (2004). 15178801 10.1126/science.1096973

[12] Vaidya, A. T. et al. Flavin reduction activates Drosophila cryptochrome. *Proceedings of the National Academy of Sciences* **110**(51), 20455–20460 (2013). 10.1073/pnas.1313336110 PMC3870761 24297896

[13] Ozturk, N. et al. Reaction mechanism of Drosophila cryptochrome. *Proceedings of the National Academy of Sciences* **108**(2), 516–521 (2011). 10.1073/pnas.1017093108 PMC3021015 21187431

[14] Lin, C. et al. Cryptochrome-timeless structure reveals circadian clock timing mechanisms. *Nature* **617**(7959), 194–199 (2023). 37100907 10.1038/s41586-023-06009-4 PMC11034853

[15] Ozturk, N. et al. Mechanism of photosignaling by Drosophila cryptochrome: role of the redox status of the flavin chromophore. *Journal of Biological Chemistry* **289**(8), 4634–4642 (2014). 24379403 10.1074/jbc.M113.542498 PMC3931024

[16] Czarna, A. et al. Structures of drosophila cryptochrome and mouse cryptochrome1 provide insight into circadian function. *Cell* **153**(6), 1394–1405 (2013). 23746849 10.1016/j.cell.2013.05.011

[17] Ozturk, N. et al. Animal type 1 cryptochromes: Analysis of the redox state of the flavin cofactor by site-directed mutagenesis. *J Biol Chem* **283**(6), 3256–3263 (2008). 18056988 10.1074/jbc.M708612200

[18] Berndt, A. et al. A novel photoreaction mechanism for the circadian blue light photoreceptor drosophila cryptochrome. *Journal of Biological Chemistry* **282**(17), 13011–13021 (2007). 17298948 10.1074/jbc.M608872200

[19] Hoang, N. et al. Human and drosophila cryptochromes are light activated by flavin photoreduction in living cells. *PLoS Biol* **6**(7), e160 (2008). 18597555 10.1371/journal.pbio.0060160 PMC2443192

[20] Ganguly, A. et al. Changes in active site histidine hydrogen bonding trigger cryptochrome activation. *Proc Natl Acad Sci USA* **113**(36), 10073–10078 (2016). 27551082 10.1073/pnas.1606610113 PMC5018803

[21] Chandrasekaran, S. et al. Tuning flavin environment to detect and control light-induced conformational switching in Drosophila cryptochrome. *Commun Biol* **4**(1), 249 (2021). 33637846 10.1038/s42003-021-01766-2 PMC7910608

[22] Zoltowski, B. D. et al. Structure of full-length Drosophila cryptochrome. *Nature* **480**(7377), 396–399 (2011). 22080955 10.1038/nature10618 PMC3240699

[23] Lin, C. et al. Circadian clock activity of cryptochrome relies on tryptophan-mediated photoreduction. *Proc Natl Acad Sci USA* **115**(15), 3822–3827 (2018). 29581265 10.1073/pnas.1719376115 PMC5899454

[24] Lin, C. et al. Mechanistic insight into light-dependent recognition of timeless by Drosophila cryptochrome. *Structure* **30**(6), 851-861.e5 (2022). 35397203 10.1016/j.str.2022.03.010 PMC9201872

[25] Dissel, S. et al. A constitutively active cryptochrome in Drosophila melanogaster. *Nat Neurosci* **7**(8), 834–840 (2004). 15258584 10.1038/nn1285

[26] Zheng, X. et al. FOXO and insulin signaling regulate sensitivity of the circadian clock to oxidative stress. *Proc Natl Acad Sci USA* **104**(40), 15899–15904 (2007). 17895391 10.1073/pnas.0701599104 PMC2000406

[27] Asher, G. et al. SIRT1 regulates circadian clock gene expression through PER2 deacetylation. *Cell* **134**(2), 317–328 (2008). 18662546 10.1016/j.cell.2008.06.050

[28] Gupta, N. & Ragsdale, S. W. Thiol-disulfide redox dependence of heme binding and heme ligand switching in nuclear hormone receptor Rev-erbβ. *Journal of Biological Chemistry* **286**(6), 4392–4403 (2011). 21123168 10.1074/jbc.M110.193466 PMC3039370

[29] Ivleva, N. B. et al. LdpA: A component of the circadian clock senses redox state of the cell. *EMBO J* **24**(6), 1202–1210 (2005). 15775978 10.1038/sj.emboj.7600606 PMC556408

[30] Ramsey, K. M. et al. Circadian clock feedback cycle through NAMPT-mediated NAD+ biosynthesis. *Science* **324**(5927), 651–654 (2009). 19299583 10.1126/science.1171641 PMC2738420

[31] Rutter, J. et al. Regulation of clock and NPAS2 DNA binding by the redox state of NAD cofactors. *Science* **293**(5529), 510–514 (2001). 11441146 10.1126/science.1060698

[32] Stoleru, D. et al. The Drosophila circadian network is a seasonal timer. *Cell* **129**(1), 207–219 (2007). 17418796 10.1016/j.cell.2007.02.038

[33] Bak, D. W. & Weerapana, E. Cysteine-mediated redox signalling in the mitochondria. *Molecular BioSystems* **11**(3), 678–697 (2015). 25519845 10.1039/c4mb00571f

[34] Wang, Y., Yang, J. & Yi, J. Redox sensing by proteins: Oxidative modifications on cysteines and the consequent events. *Antioxidants & Redox Signaling* **16**(7), 649–657 (2011). 21967570 10.1089/ars.2011.4313

[35] Schmalen, I. et al. Interaction of circadian clock proteins CRY1 and PER2 is modulated by zinc binding and disulfide bond formation. *Cell* **157**(5), 1203–1215 (2014). 24855952 10.1016/j.cell.2014.03.057

[36] Levy, C. et al. Updated structure of Drosophila cryptochrome. *Nature* **495**(7441), E3–E4 (2013). 23518567 10.1038/nature11995 PMC3694752

[37] Hemsley, M. J., et al. Linear motifs in the C-terminus of D. melanogaster cryptochrome. *Biochemical and Biophysical Research Communications* **355**(2), 531–537 (2007). 10.1016/j.bbrc.2007.01.189 17306225

[38] Damulewicz, M. & Mazzotta, G. M. One actor, multiple roles: The performances of cryptochrome in drosophila. *Front Physiol* **11**, 99 (2020). 32194430 10.3389/fphys.2020.00099 PMC7066326

[39] Krishnan, B. et al. A new role for cryptochrome in a Drosophila circadian oscillator. *Nature* **411**(6835), 313–317 (2001). 11357134 10.1038/35077094

[40] Collins, B. et al. Drosophila CRYPTOCHROME is a circadian transcriptional repressor. *Current Biology* **16**(5), 441–449 (2006). 16527739 10.1016/j.cub.2006.01.034

[41] Froy, O., Chang, D. C. & Reppert, S. M. Redox potential: Differential roles in dCRY and mCRY1 functions. *Current Biology* **12**(2), 147–152 (2002). 11818067 10.1016/s0960-9822(01)00656-x

[42] Vieira, J. et al. Human cryptochrome-1 confers light independent biological activity in transgenic drosophila correlated with flavin radical stability. *PLoS ONE* **7**(3), e31867 (2012). 22427812 10.1371/journal.pone.0031867 PMC3299647

[43] Emery, P. et al. Drosophila CRY is a deep brain circadian photoreceptor. *Neuron* **26**(2), 493–504 (2000). 10839367 10.1016/s0896-6273(00)81181-2

[44] Zoltowski, B. D. et al. Structure of full-length Drosophila cryptochrome. *Nature* **480**(7377), 396–399 (2012). 10.1038/nature10618 PMC3240699 22080955

[45] Vaidya, A. T. et al. Flavin reduction activates Drosophila cryptochrome. *Proc Natl Acad Sci USA* **110**(51), 20455–20460 (2013). 24297896 10.1073/pnas.1313336110 PMC3870761

[46] Nakashima, H. Effects of respiratory inhibitors on respiration, ATP contents, and the circadian conidiation rhythm of Neurospora crassa. *Plant Physiology* **76**(3), 612–614 (1984). 16663893 10.1104/pp.76.3.612 PMC1064342

[47] Rutledge, E. M., Mongin, A. A. & Kimelberg, H. K. Intracellular ATP depletion inhibits swelling-induced d-[3H]aspartate release from primary astrocyte cultures. *Brain Research* **842**(1), 39–45 (1999). 10526093 10.1016/s0006-8993(99)01805-3

[48] Li, N. et al. A systematic assessment of mitochondrial function identified novel signatures for drug-induced mitochondrial disruption in cells. *Toxicological Sciences* **142**(1), 261–273 (2014). 25163676 10.1093/toxsci/kfu176

[49] Wyatt, C. N. & Buckler, K. J. The effect of mitochondrial inhibitors on membrane currents in isolated neonatal rat carotid body type I cells. *The Journal of Physiology* **556**(1), 175–191 (2004). 14724184 10.1113/jphysiol.2003.058131 PMC1664886

[50] Barile, M. et al. The riboflavin/FAD cycle in rat liver mitochondria. *European Journal of Biochemistry* **267**(15), 4888–4900 (2000). 10903524 10.1046/j.1432-1327.2000.01552.x

[51] Pallotta, M. L. et al. Saccharomyces cerevisiae mitochondria can synthesise FMN and FAD from externally added riboflavin and export them to the extramitochondrial phase. *FEBS Lett* **428**(3), 245–249 (1998). 9654142 10.1016/s0014-5793(98)00544-4

[52] Kobayashi, K. et al. Characterization of photolyase/blue-light receptor homologs in mouse and human cells. *Nucleic Acids Research* **26**(22), 5086–5092 (1998). 9801304 10.1093/nar/26.22.5086 PMC147960

[53] Kleine, T., Lockhart, P. & Batschauer, A. An Arabidopsis protein closely related to Synechocystis cryptochrome is targeted to organelles. *The Plant Journal* **35**(1), 93–103 (2003). 12834405 10.1046/j.1365-313x.2003.01787.x

[54] Hemsley, M. J., et al., Linear motifs in the C-terminus of D. melanogaster cryptochrome. *Biochem Biophys Res Commun* **355**(2), 531–7 (2007). 10.1016/j.bbrc.2007.01.189 17306225

[55] Diekmann, C. & Brody, S. Circadian rhythms in Neurospora crassa: oligomycin-resistant mutations affect periodicity. *Science* **207**(4433), 896–898 (1980). 6444467 10.1126/science.6444467

[56] Brody, S. Circadian rhythms in Neurospora crassa: the role of mitochondria. *Chronobiol Int* **9**(3), 222–230 (1992). 1535289 10.3109/07420529209064531

[57] Sathyanarayanan, S. et al. Identification of novel genes involved in light-dependent CRY degradation through a genome-wide RNAi screen. *Genes Dev* **22**(11), 1522–1533 (2008). 18519643 10.1101/gad.1652308 PMC2418588

