# Supplementary material for: Mitochondrial inhibitors reveal roles of specific respiratory chain complexes in CRY-dependent degradation of TIM
Source: Sci Rep. 2024 Oct 30;14:26051. doi: 10.1038/s41598-024-77692-0 (PMC11522321; doi:10.1038/s41598-024-77692-0)
Supplement: Supplementary file 1 — Supplementary Material 1 [file 41598_2024_77692_MOESM1_ESM.docx]

**Supplemental figures**

**Mitochondrial inhibitors reveal roles of specific respiratory chain complexes in CRY-dependent degradation of TIM**

Xiangzhong Zheng* 1,2, Dechun Chen 1, Brian Zoltowski 3, Amita Sehgal* 1,4

1. Department of Neuroscience, University of Pennsylvania Perelman School of Medicine, Philadelphia, PA 19104
2. Department of Biology, Indiana University, Bloomington, IN 47405
3. Department of Chemistry, Southern Methodist University, Dallas, TX 75275
4. Howard Hughes Medical Institute

*Corresponding author: [samzheng@iu.edu](mailto:samzheng@iu.edu); [amita@pennmedicine.upenn.edu](mailto:amita@pennmedicine.upenn.edu)


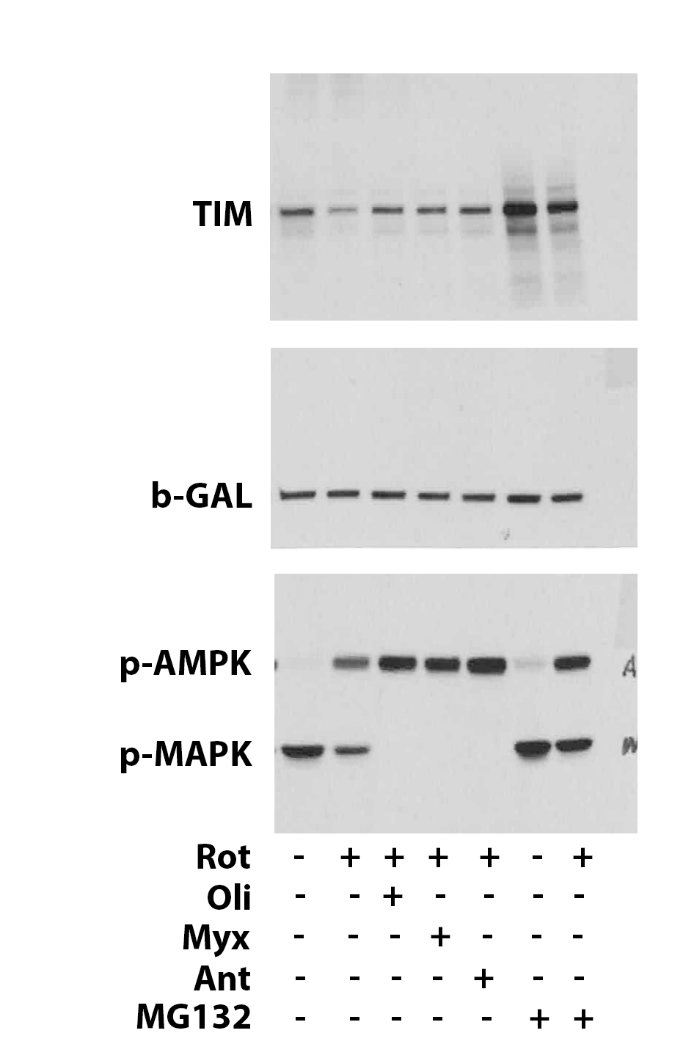


Figure S1. The effect of rotenone is blocked by complex III and complex V inhibitors. Complex III inhibitors (antimycin A, myxothiazol) and complex V inhibitor (oligomycin) rescued rotenone-mediated degradation of TIM in darkness. Mitochondrial inhibitors were used at 2 µM. The proteasome inhibitor MG132 was used at 100 µM. Original blots were cut to probe TIM and p-AMPK/p-MAPK separately.



Figure S2. Full length blots for Figure 1E and Figure 3C. phospho-AMPK and phospho-MAPK were probed but not used in this study. Right panel bottom blots were exposed longer to visualize CRY^m^ band. Mitochondrial inhibitors oligomycin (Oli) and antimycin A (Ant) did not block CRY^m^ mediated TIM degradation. v: EtOH; wt: wildtype; b: cry^b^; m: cry^m^; bm: cry^bm^.. Original blots were cut to probe TIM and CRY/JET separately.


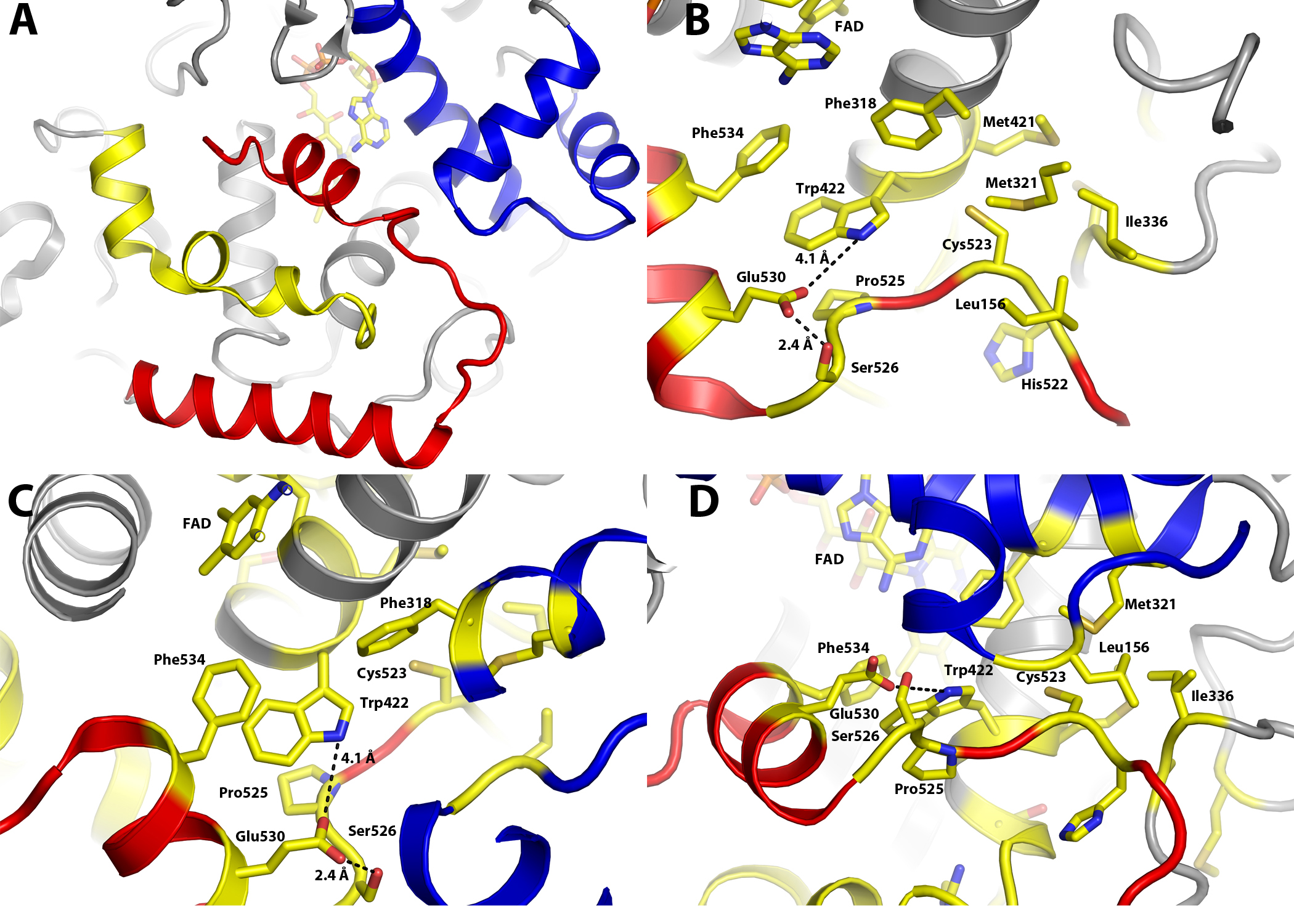


Figure S3: Structural modeling of CRY C-terminal and Turn Motif. Structural analysis was conducted using PDBID 4GU5 [[1](#_ENREF_1)]. All figures were generated using PYMOL (The PyMOL Molecular Graphics System, Version 1.8 Schrödinger, LLC). (**A**) The C-terminal tail (CTT) and coiled-coil helix (red) is located adjacent to the C-terminal lid (yellow) and helical-interface (blue). The turn motif connects the CTT and coiled-coil helix and makes extensive contacts with the helical interface (see C,D). (**B**) The turn motif forms a sulfur-rich loop that may relay photochemistry or conformational responses from the FAD through the CTT. F534 lies adjacent to the active site FAD and makes close contacts with W422 which is anchored in a hinge region in the turn motif. E530 forms a strong H-bond with Ser526, but is too far from W422 indole to form H-bonding interactions. C523 lies in a hydrophobic pocket to anchor the turn motif. (**C,D**) Close up of contacts between turn motif and helical interface to form a hydrophobic pocket containing W422 and C523.

1. Levy, C., et al., *Updated structure of Drosophila cryptochrome.* Nature, 2013. **495**(7441): p. E3-E4.


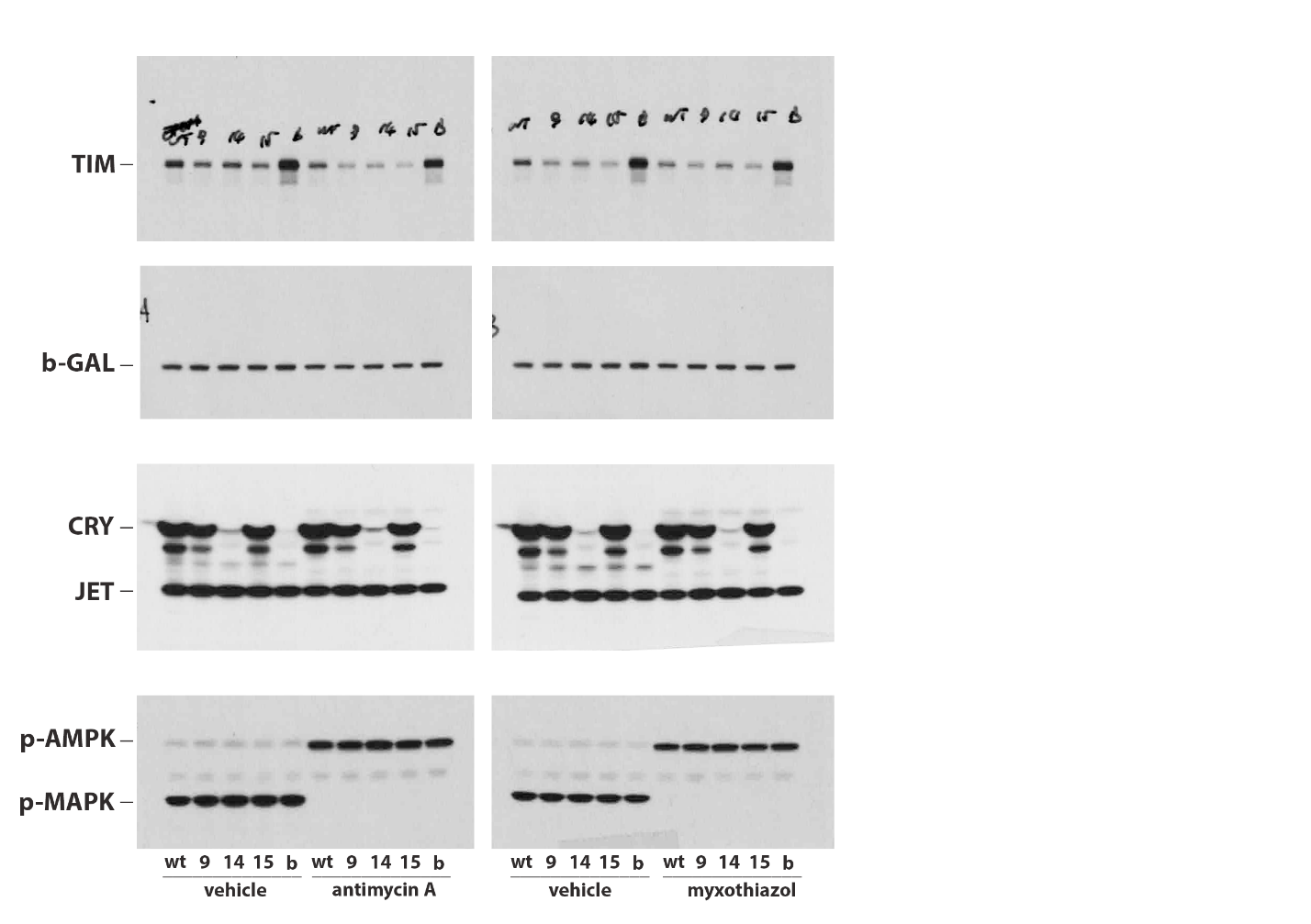


Figure S4. Full length blots for Figure 2 panel C. Complex III inhibitors antimycin A and myxothiazol did block the effect of CRY CTT mutants (9: C523S, 14: E530Q, 15: F534L) on TIM. Original blots were cut to probe TIM and CRY/JET separately.





Figure S5. Expression levels of *cry* constructs in cell culture. (A) pCDNA3.1-*cry* plasmids were transfected into HEK293T cells. 48 h after transfection, cells lysates were processed for Western blotting and probed with anti-CRY and anti-ACTIN antibodies. All mutant constructs have reduced expression levels compared to wildtype CRY. (B) pIZ-m*Cry1*-V5 plasmids were transfected into Drosophila S2 cells. 48 h after transfection, cells were treated with DMSO or rotenone (2 uM) for 6 h. Cell lysates were processed for Western blotting and probed with anti-V5 and MAPK antibodies. Full-length scan is shown in Figure S12 and Figure S13.


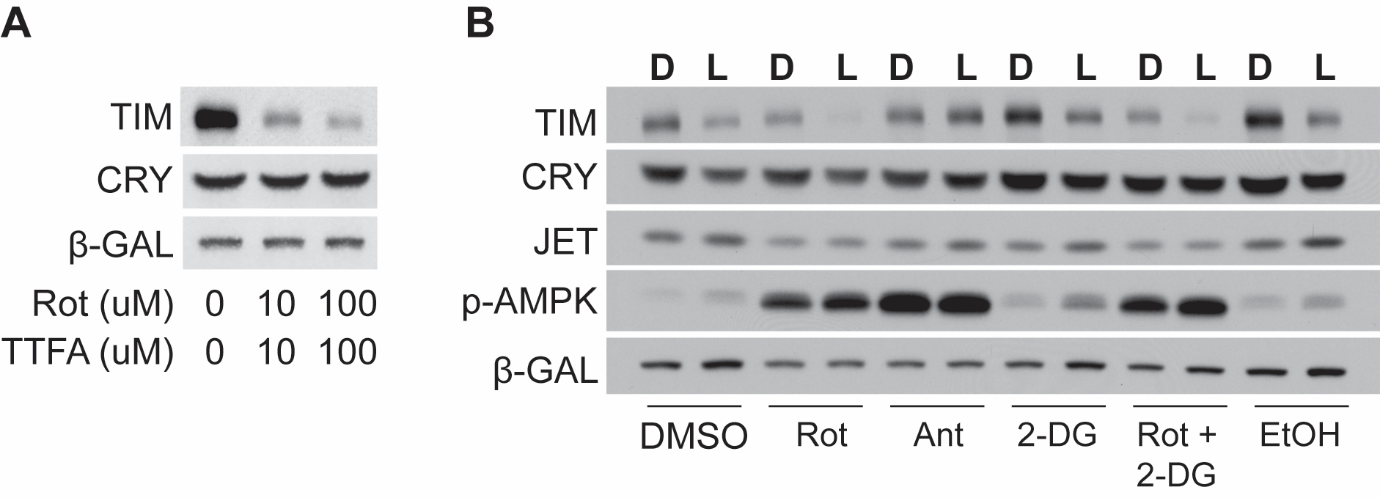


Figure S6. Effect of mitochondrial inhibitors and glycolytic inhibitor 2-deoxyglucose (2-DG) on TIM stability. *Drosophila* S2R^+^ cells were transfected with the following plasmids: pIZ-*tim*, pIZ-*myc-cry*, pIZ-Flag-*jet*, and pAc-β*Gal*-V5. Cell culture plates were wrapped with double-layer aluminum foil to prevent light exposure. (**A**) Cells were treated with vehicle or inhibitors in dark for 2 h, then processed for cell lysis and Western blotting assay, or (**B**) for 1 h in dark, then exposed to light for 20 min, followed by 30 min incubation in dark, then processed for cell lysis and Western blotting assay. Drugs were used at the following concentrations: Rot (1 uM); Ant (0.1 uM); 2-DG (100 mM). Full-length scan is shown in Figure S14 for panel A and Figure S15 for panel B.


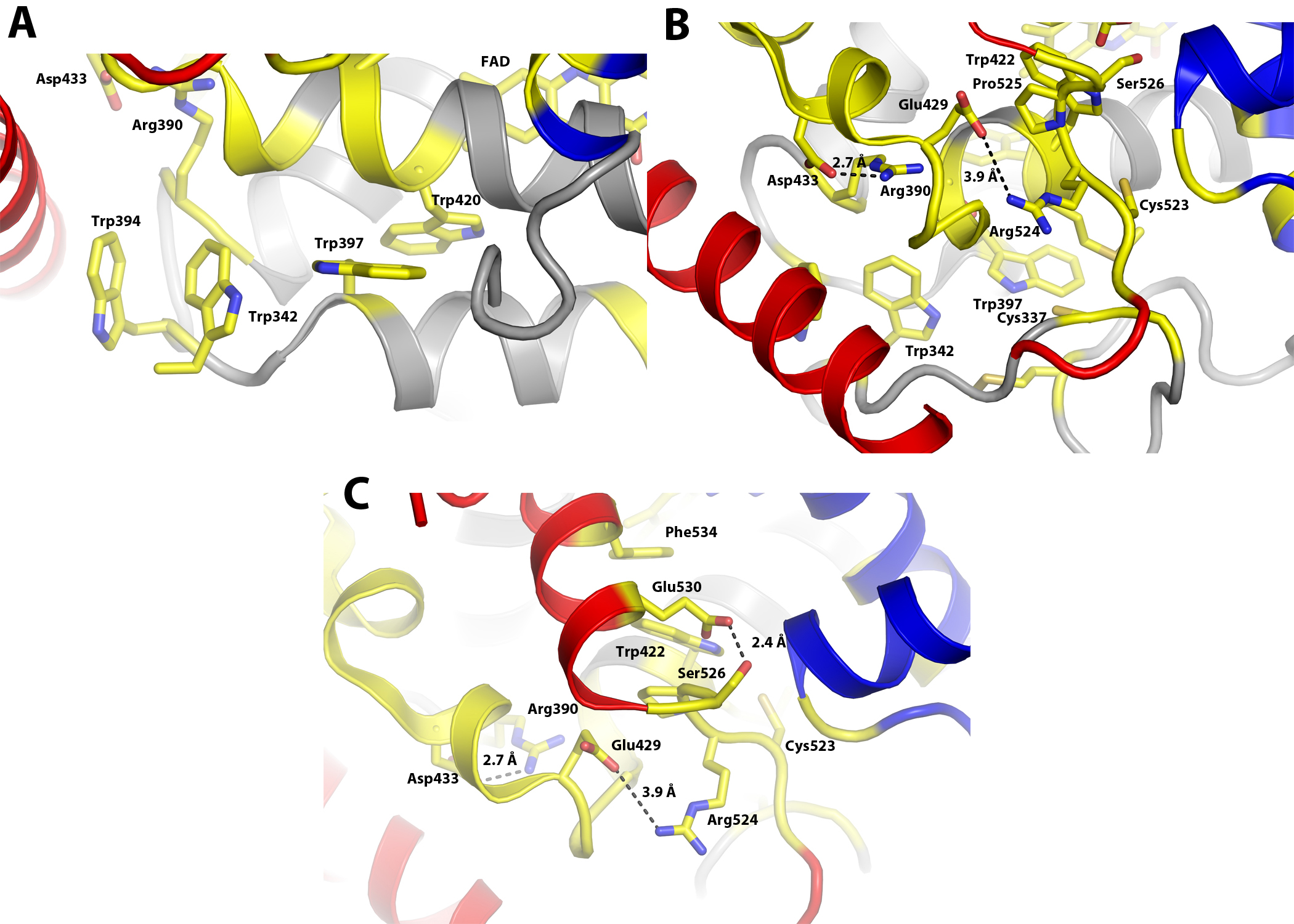


Figure S7: Modeling of signal propagation in dCRY. Structural analysis was conducted using PDBID 4GU5 [[1](#_ENREF_1)]. All figures were generated using PYMOL (The PyMOL Molecular Graphics System, Version 1.8 Schrödinger, LLC). (**A**) Photoactivation of dCRY has been proposed to proceed through the Trp triad composed of W420/W397/W342. An additional W394 is likely the terminal Trp residue and lies adjacent to the coiled-coil helix (red). (**B**) Examination of interactions between the Trp-triad, coiled coil helix (red) and the turn motif. Several salt bridges interact between the structural elements and are adjacent to the Trp triad. D433 forms a strong salt interaction with R390 that acts as a cap to stabilize the helical dipole containing W420 (proximal electron donor). A neighboring possible salt bridge E429-R534 between the C-terminal lid and CTT may form following photoexcitation. Alternatively these motifs may be involved in shifting of salt bridges between key elements. (**C**) Three possible salt bridges involving key structural elements. E530 forms a strong H-bond with S526. Neighboring E439 and R524 are too far apart for H-bonding or salt interactions. However, R390-D433 readily forms between the C-terminal lid and the capping Arg. Photoexcitation may involve rearrangement of these interactions.

1. Levy, C., et al., *Updated structure of Drosophila cryptochrome.* Nature, 2013. **495**(7441): p. E3-E4.

**Full-length blots for Figures 1, Figure 2, Figure 3, Figure S5, Figure S6:**





Figure S8. Full-length blots related to Figure 1 panel A. HSP70 was probed but not used in this study. Original blots were cut to probe TIM and CRY separately.





Figure S9. Full-length blots related to Figure 1 panel C.





Figure S10. Full-length blots related to Figure 2 A. Original blots were cut to probe TIM and CRY separately.





Figure S11. Full-length blots related to Figure 3 panel A. Original blots were cut to probe TIM (not used in this figure) and CRY/JET separately.


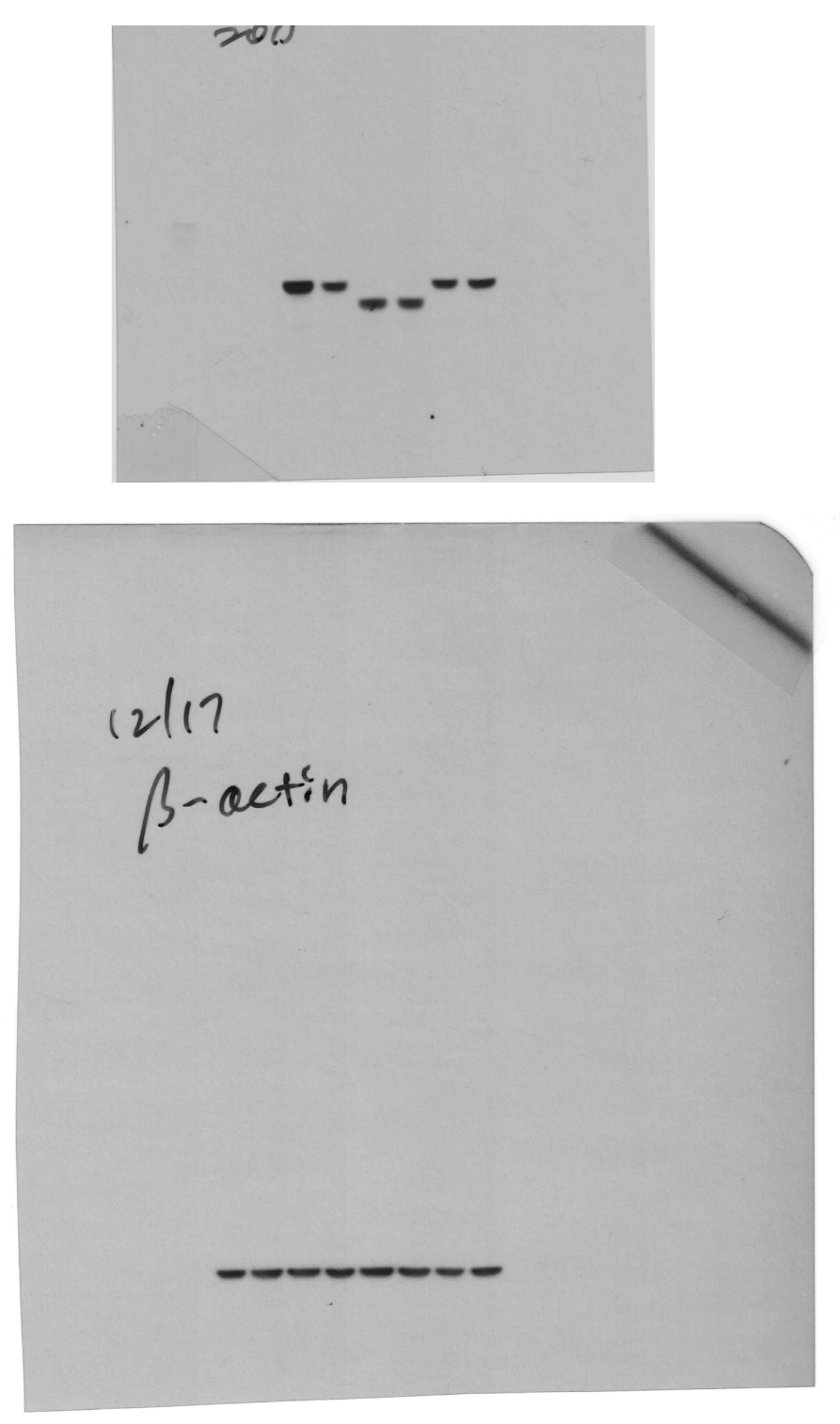


Figure S12. Full-length scan for Figure S5 panel A. Bands in orange box area were used for Figure S5A.

Figure S13. Full-length scan for Figure S5 panel B. Bands in orange box area were used for Figure S5B.


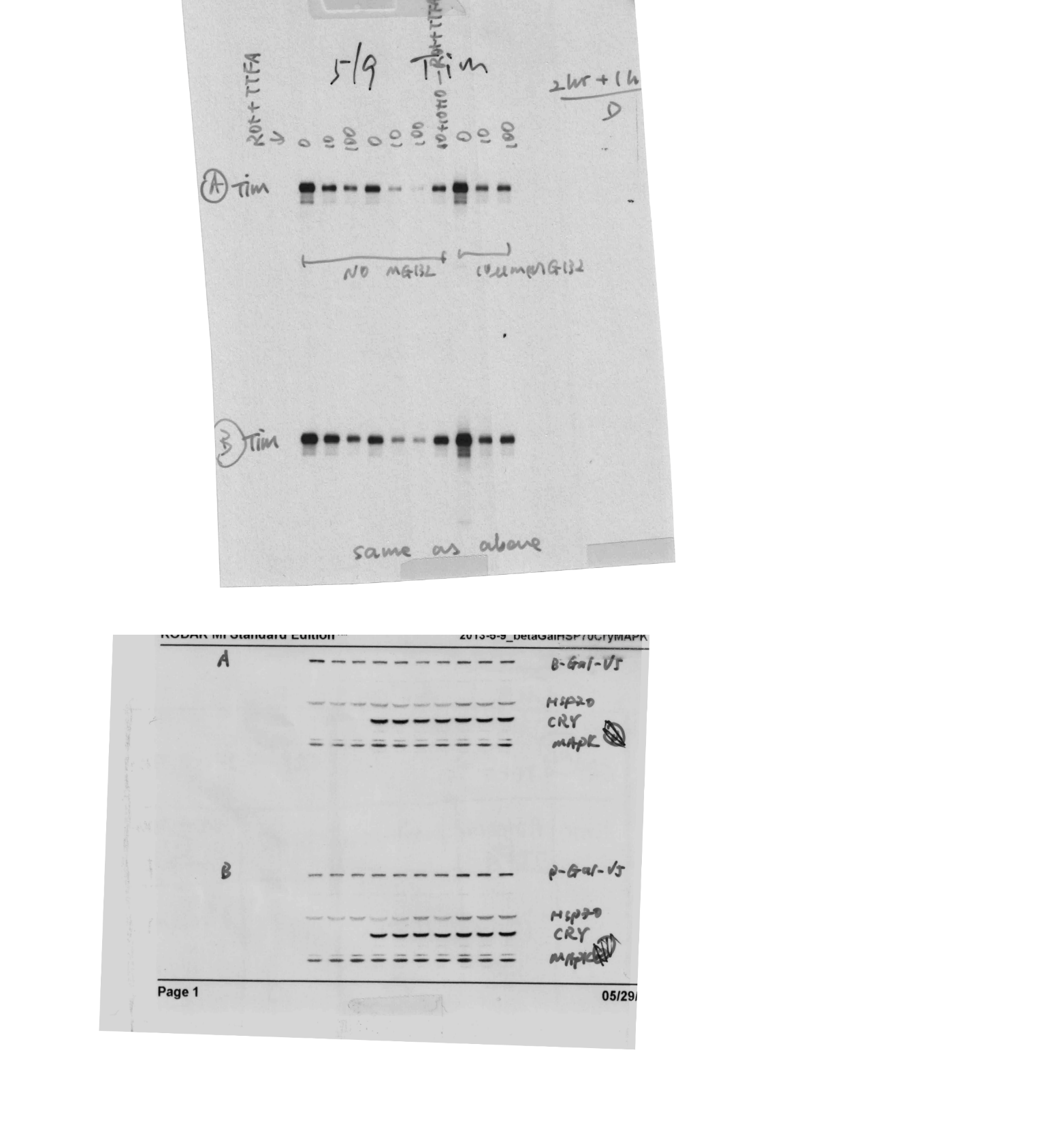


Figure S14. Full-length scan for Figure S6 panel A. Bands in orange box area were used for Figure S6A. Original blots were cut to probe TIM and CRY/MAPK separately.


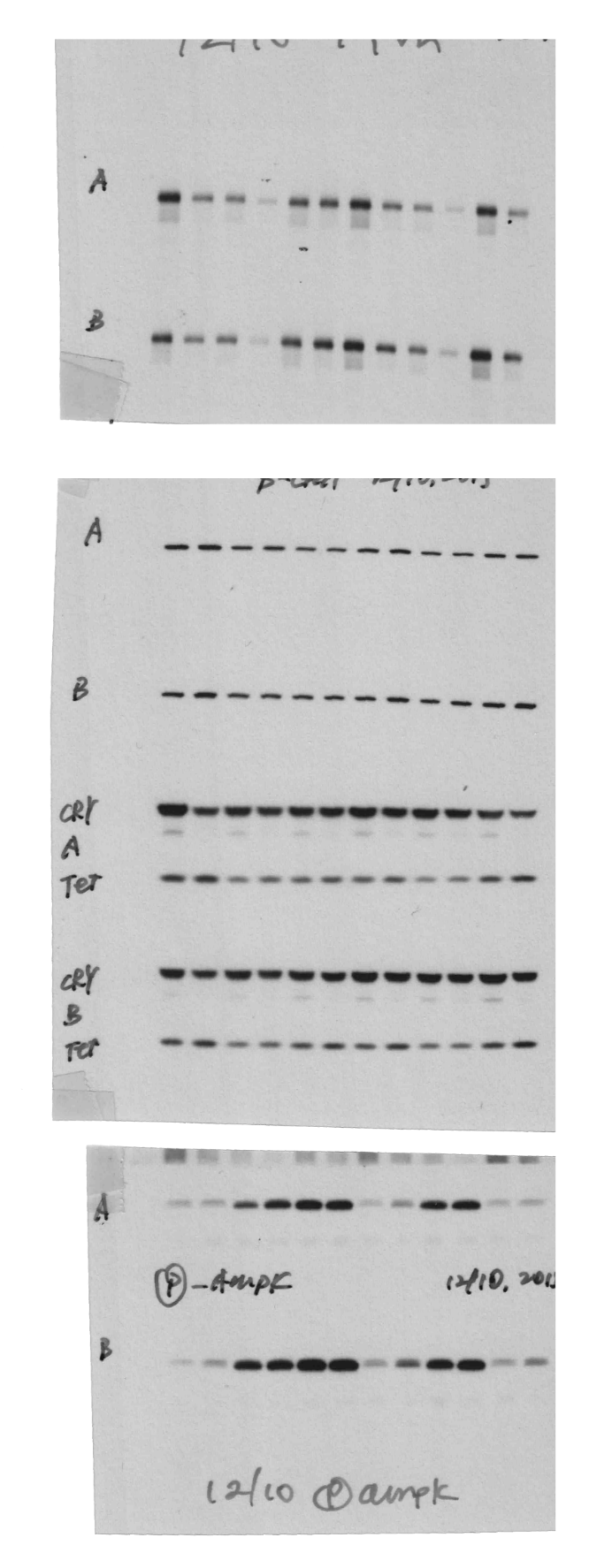


Figure S15. Full-length scan for Figure S6 panel B. Bands in orange box area were used for Figure S6B. Original blots were cut to probe TIM and CRY/JET separately.
